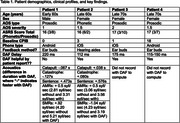# Evaluating the impact of delayed auditory feedback in primary progressive apraxia of speech: acoustic, perceptual, and patient reported outcomes

**DOI:** 10.1002/alz70858_100319

**Published:** 2025-12-24

**Authors:** Rene L Utianski, Joseph R Duffy, Gabriela Meade, Ashley D Bachman, Hugo Botha

**Affiliations:** ^1^ Mayo Clinic, Rochester, MN, USA; ^2^ Department of Neurology, Mayo Clinic, Rochester, MN, USA

## Abstract

**Background:**

Primary progressive apraxia of speech (PPAOS) is a neurodegenerative disorder affecting speech motor planning/programming. Current interventions for PPAOS are limited and often adapted from treatments for nondegenerative AOS, which rely on intensive practice to maintain speech accuracy for practiced items. Systematic interventions for PPAOS are scarce, and no studies have explored differential effects across AOS subtypes. PPAOS subtypes include those with predominant phonetic (articulatory distortions and substitutions) or prosodic (slow speech rate, segmentation) speech changes. These subtypes likely differ in their neural underpinnings and may respond variably to intervention. Delayed auditory feedback (DAF), which introduces a slight delay in hearing one's speech, has shown promise in altering speech rate and fluency for stuttering and hypokinetic dysarthria, though its effects on stroke‐related AOS have been mixed. Recent studies suggest DAF can increase speech rate in patients with AOS in the context of nonfluent/agrammatic primary progressive aphasia. We hypothesized DAF would improve speech clarity and fluency in PPAOS, with subtype‐specific effects: phonetic PPAOS may see slower, more accurate speech, while prosodic PPAOS may produce faster, less segmented speech.

**Method:**

Four PPAOS patients (3 prosodic, 1 phonetic) completed a web‐based speech battery with and without DAF. Feedback delay was calibrated individually using the DAF Pro app. Speech metrics were analyzed acoustically, and blinded perceptual ratings of deviant speech features were conducted.

**Results:**

Key data are summarized in Table 1. Three out of four patients found the DAF helpful and two re‐recorded with DAF to allow for quantitative analysis. Patient 1 produced words faster with DAF but had slower total sentence production times; he did not find it helpful. Patient 2 spoke slower with DAF. Both patients produced AMRs faster with DAF, while SMRs were faster for Patient 1 and slower for Patient 2. Blinded perceptual judgments of speech clarity and prosody revealed mixed effects of DAF across participants.

**Conclusions:**

Preliminary results suggest DAF may differentially benefit phonetic and prosodic PPAOS, particularly in sentences compared to word production. Further research is needed to assess clinical significance, refine DAF calibration, and explore patient‐perceived improvements with guided practice in a larger, more diverse sample.